# Short versus standard treatment with pegylated interferon alfa-2A plus ribavirin in patients with hepatitis C virus genotype 2 or 3: the cleo trial

**DOI:** 10.1186/1471-230X-10-21

**Published:** 2010-02-19

**Authors:** Fabrizio Mecenate, Adriano M Pellicelli, Giuseppe Barbaro, Mario Romano, Angelo Barlattani, Ettore Mazzoni, Maria Elena Bonaventura, Lorenzo Nosotti, Pasquale Arcuri, Antonio Picardi, Giorgio Barbarini, Cecilia D'Ambrosio, Amerigo Paffetti, Arnaldo Andreoli, Fabrizio Soccorsi, the Club Epatologi Ospedalieri (CLEO) Group

**Affiliations:** 1 Liver Unit Villa Betania Hospital, Rome, Italy; 2 Liver Unit San Camillo Forlanini Hospital, Rome, Italy; 3 Liver Unit Pertini Hospital, Rome, Italy; 4 Department of Medical Pathophysiology, Policlinico Umberto I, University of Rome La sapienza Rome, Italy; 5 Liver Unit San Giacomo Hospital, Rome, Italy; 6 Liver Unit Policlinico Casilino, Rome, Italy; 7 Division of Infectious Disease San Camillo De Lellis Hospital, Rieti, Italy; 8 IRCCS San Gallicano Hospital, Rome, Italy; 9 Liver Unit San Giovanni Hospital, Rome, Italy; 10 Liver Unit Campus Biomedico University, Rome, Italy; 11 Liver Unit IRCCS San Matteo, Pavia, Italy; 12 Division of Infectious disease Policlinico Umberto I Rome, Italy

## Abstract

**Background:**

In patients with chronic hepatitis C virus (HCV) genotype 2 or 3, 24 weeks' treatment with pegylated interferon alfa (PEG-IFN-alpha) and ribavirin induces a sustained virological response (SVR) in almost 80% of cases. Evidence suggests that a similar response rate may be obtained with shorter treatment periods, especially in patients with a rapid virological response (RVR). The aim of this study was to compare the efficacy of 12 or 24 weeks of treatment in patients with chronic HCV genotype 2 or 3 and to identify patients suitable for 12 weeks treatment.

**Methods:**

Two hundred and ten patients received PEG-IFN-alpha-2a (180 ug/week) and ribavirin (800-1200 mg/day) for 4 weeks. Patients with a RVR (HCV RNA not detectable) were randomized (1:1) to either 12 (group A1) or 24 (group A2) weeks of combination therapy. Patients without a RVR continued with 24-weeks' combination therapy (group B). HCV RNA was monitored at weeks 4, 8, 12, and 24, and at week 24 post-treatment.

**Results:**

At study end, end of treatment response (ETR) was observed in 62 (86%) patients of group A1 and in 55 (77%) patients of group A2 (p < 0.05) Relapse rate was 3% each in groups A1 and A2, and 6% in group B. Among patients with a HCVRNA test 24 weeks after the end of treatment, SVR was observed in 60 (83%) of group A1 patients and in 53 (75%) of group A2 patients. Rapid virological response, low baseline HCV RNA levels, elevated alanine aminotransferase levels and low fibrosis score, were the strongest covariates associated with SVR, independent of HCV genotype. No baseline characteristic was associated with relapse.

**Conclusion:**

In HCV patients with genotype 2 or 3, 12-week combination therapy is as efficacious as 24-week therapy and several independent covariates were predictive of SVR.

**Trial registration:**

Trial number ISRCTN29259563

## Background

Pegylated interferon alpha (PEG-IFN-α) and ribavirin administered in combination for a period of 24 or 48 weeks ensure a sustained virological response (SVR) in most patients with chronic hepatitis C virus (HCV) infection and genotype 2 or 3 [1-3]. A shorter course of therapy with PEG-IFN-α-2b and ribavirin has been shown to be as effective as a 24-week course for patients with HCV genotype 2 or 3, especially those with a rapid virological response (RVR) [4,5]. Upon initiation of interferon therapy, there is a rapid decline in viral load, reflecting the efficiency of interferon-dependent inhibition of the production of the virus, its release, or both [4]. This rapid decline is followed by a slower decrease in viral load that is dependent on the rate of death of infected cells and is estimated to vary from 1.7 days to more than 70 days [6]. The rate of decline in the second phase is eight times faster in patients with genotype 2 or 3 compared with patients with genotype 1 [7,8]. This difference suggests that patients with HCV genotype 2 or 3 infection may need shorter courses of therapy than the regimens currently recommended [4]. Changes in viremia levels over the first weeks of therapy correlate with the likelihood of the eradication of HCV, and undetectable viral levels at week 12 are predictive of a response after 48 weeks of therapy [9,10]. Preliminary clinical data have shown that in patients with HCV genotype 2 or 3 in whom HCV RNA is not detectable after 4 weeks of therapy, 12 weeks of treatment with PEG-IFN-α-2a or 2b and ribavirin in combination may be as effective as the recommended course of 24 weeks [4,11-13]. This is in agreement with the hypothesis based on viral kinetics data [14]. We therefore conducted a randomized clinical trial to assess whether a 12-week regimen of a combination of PEG-IFN-α-2a and ribavirin was as efficacious as a 24-week regimen in patients with HCV genotype 2 or 3.

## Methods

### Objective and hypothesis

The following hypothesis was tested: in patients with HCV genotype 2 or 3 and with an RVR, 12 weeks of treatment with PEG-IFN-α-2a at a dose of 180 μg subcutaneously once weekly and oral ribavirin, at a dosage of 800-1200 mg/day is comparable to a 24 weeks treatment. Futhermore, it was hypothesized that a 12 weeks treatment had fewer side effects respect to 24 weeks course.

### Patient selection

Patients were eligible for inclusion if they were HCVRNA positive, had HCV genotype 2 or 3, had elevated alanine aminotransferase (>40 UI/L) at least 8 months prior to study entry and had an histologically proven chronic HCV hepatitis. Patients were excluded if they were known to have injected drugs or alcohol abuse (>40 g ethanol/day) within the 6 months prior to study entry; had poorly controlled psychiatric illness; had decompensated cirrhosis; were positive for human immunodeficiency antibody virus or positive for hepatitis B surface antigen. Additional criteria for exclusion were: pregnancy, lactation, impaired renal function, and other concurrent medical conditions of the liver different from HCV infection.

### Study design

**Figure 1:**
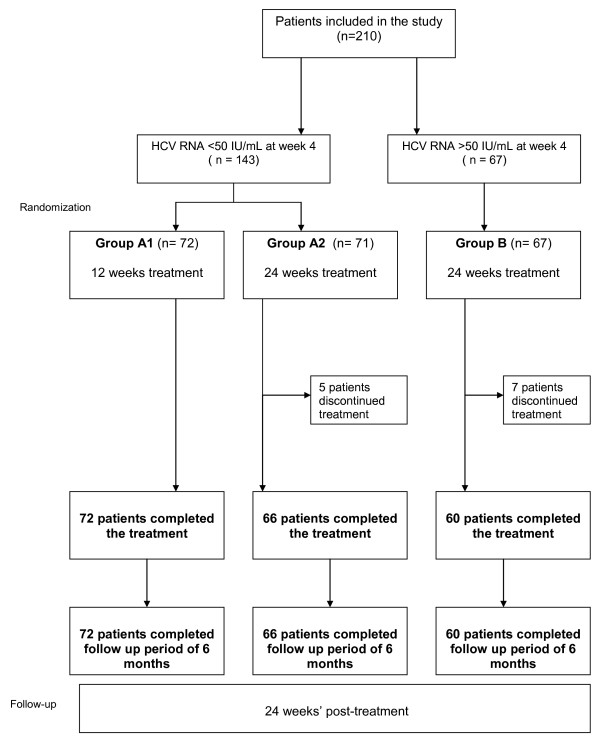
**CLEO study design**. During the treatment phase, all patients received PEG-IFN-α-2a 180 μg/week + ribavirin 800-1200 mg/day. PEG-IFN = pegylated interferon

Patients fulfilling the selection criteria received in an open-label fashion PEG-IFN-α-2a at a dose of 180 μg subcutaneously once weekly and oral ribavirin, at a dosage of 800 mg/day (for those with a weight of < 65 kg), 1000 mg/day (for those with a weight between 65 and 85 kg) or 1200 mg/day (for those with a weight > 85 Kg). Patients with Rapid Virological response (RVR) defined as HCVRNA <50 UI/ml after 4 weeks of treatment, were randomly assigned in a 1:1 ratio to receive a treatment either for 12 (Group A1) or 24 (Group A2) weeks. Patients without RVR were treated for a standard period of 24 weeks (Group B) (Figure 1).

### Study endpoints

The primary efficacy endpoint was SVR which was defined as undetectable plasma HCVRNA (<50 UI/ml) 24 weeks after the end of treatment. All the patients who withdrew from the study and with HCV-RNA detectable at 24 weeks after the end of the treatment, were defined as non-responders. Patients experiencing adverse events during the study had their dose of ribavirin reduced to 800 mg/day. Hematological biochemical testing in participants were performed, on an outpatient basis, at weeks 4, 8, 12, 16 and 24 during treatment, and 24 weeks after the end of treatment. Dose reduction or suspension of study drugs was considered if serious adverse effects occurred. Adherence to treatment was assessed using patient diaries.

### Virology

HCV-RNA determination was performed with the COBAS Amplicore HCV test (lower detection level 50 UI/ml). HCV genotyping was performed with the use of a hybridization technique (Innolipa HCV Immunogenetics) All quantifications were performed at one central laboratory. HCV-RNA analysis was performed at week 4, at the end of treatment (12 or 24 weeks) and 24 weeks after the end of treatment.

### Liver Biopsy

Liver biopsies were obtained from all patients within 20 months prior to study entry. Only biopsy with a length exceeding 1 cm and containing more than 6 portal tracts were evaluated. All the biopsy were graded by two indipendent observers and the evaluation was performed according to the Ishak score [15].

### Statistical analysis

The noninferiority margin was set at 20% between Groups A1 and A2. To obtain 80% statistical power with alpha level = 0.05, and Beta = 0.20, approximately 70 patients per treatment group were necessary. Initial enrollment plans included 210 patients. Intention to treat analysis was performed. All data were expressed as the median and range for discrete variables and as counts and percentage for qualitative variables. Continuous data were analysed using the Mann-Whitney test and the Wilcoxon signed rank test for paired analyses. Categorical data were analysed using the chi-square test with the Yates's correction and the Fisher's exact test. The relationship between pretreatment independent covariates and the rate of SVR was examined by stepwise logistic regression analysis with related odds ratio and 95% confidence intervals (95% CI). For logistic regression analysis pretreatment covariates of all patients enrolled in the study were considered.

### Ethics

The study was approved by a central ethic committee (S. Camillo Hospital, Rome, Italy) and was conducted according to the guidelines of the International Conference on Harmonization for Good Clinical Practice. All patients gave their written informed consent prior to treatment.

## Results

Enrollment in the CLEO Centers started in July 2006, and the trial ended in January 2008. Two hundred and ten outpatients were consecutively enrolled. Baseline characteristics of the patients enrolled for the study are reported in Table 1.

**Table 1:** Baseline characteristics of 210 patients enrolled for the study

Characteristic	
Male, n (%)	170 (81)

Age, years	43 (20-68)

Body mass index, kg/m^2^	24 (21-32)

V iral Load	
-<400.000 UI/ml	104(49)
->400.000 UI/ml	106(51)

Route of Trasmission	
-Intravenous drug use	142(68)
-Transfusion	26(12)
- Unknown	42(20)

Alanine aminotransferase, IU/L	147 (96-255)

HCV genotype 2, n (%)	116 (55)

HCV genotype 3, n (%)	94 (45)

Cirrhosis (Ishak stage 5-6) n (%)	21 (10)

Bridging fibrosis (Ishak stage 3-4) n (%)	41 (19)

All values are expressed as median (range) unless otherwise specified.

### RVR

One hundred and forty-three patients (68%); 79 with genotype 2 and 64 with genotype 3 had a RVR to treatment at week 4 (Group A). Of those, 72 were randomized to receive combination treatment for 12 weeks (Group A1) and 71 to receive treatment for 24 weeks (Group A2). Sixty-seven patients (32%) did not achieve a RVR at week 4 and continued with combination therapy for 24 weeks (Group B). The characteristics of patients with RVR are reported in Table 2.

**Table 2:** Patients characteristics according to achievement of rapid virological response.

Characteristic	Group A (n = 143)	Group B (n = 67)	P value
Male, no. (%)	116 (81)	54 (80)	NS

Age, years	42 (20-68)	45 (37-66)	NS

Body mass index, kg/m^2^	24 (22-32)	25 (21-30)	NS

V iral Load			
-<400.000 UI/ml	83(58)	22(33)	< 0.01
->400.000 UI/ml	60(42)	45(67)	< 0.01

Route of Trasmission			
-Intravenous drug use	94(66)	48(72)	NS
-Transfusion	17(12)	9 (13)	NS
- Unknown	32(22)	10(15)	NS

Alanine aminotransferase, IU/L	150(98-255)	145 (96-208)	NS

HCV genotype 2, n (%)	79 (55)	37 (55)	NS

HCV genotype 3, n (%)	64 (45)	30 (45)	NS

Cirrhosis (Ishak stage 5-6) n(%)	13 (9)	8 (12)	NS

Bridging fibrosis (Ishak stage 3-4) n(%)	26 (18)	15 (22)	NS

All values are expressed as median (range) unless otherwise specified.

### SVR

According to intention to treat analysis, ETR was observed in 62 patients (86%) of group A1, in 55 patients (77%) of group A2 and in 37 patients (55%) of group B. During the follow-up period, relapse was observed in 2 patients of Group A1 (one each with genotype 2 and genotype 3), in 2 patients of Group A2 (both with genotype 3) and in 4 patients of Group B (one with genotype 2 and three with genotype 3). Patients who relapsed, after a wash-out period of 6 months, received treatment for a further 24 weeks and, of these, 5 patients (2 of Group A1, 1 of Group A2, and 2 of Group B) subsequently showed a SVR. Two of these patients had genotype 2 and 3 had genotype 3. According to intention to treat analysis, SVR was observed in 60 patients of Group A1 (83%; 32 with genotype 2 and 28 with genotype 3), in 53 patients of Group A2 (75%; 31 with genotype 2 and 22 with genotype 3), and in 33 patients of Group B (49%; 15 with genotype 2 and 18 with genotype 3) (Table 3).

**Table 3:** Virological response observed in the study groups 24 weeks after the end of the treatment

Virological response	Group A1 (n = 72)	Group A2 (n = 71)	Group B (n = 67)
Sustained virological response n.(%)	60 (83)*	53(75)†	33 (49)

Non sustained virological response n.(%)	12 (17)	18 (25)	34 (51)‡

*P < 0.001 compared with Group B

† P < 0.01 compared with Group B

‡ P < 0.001 compared with Group A1. P < 0.01 compared with Group A2.

### Factors associated with SVR

Stepwise logistic regression analysis showed that RVR (odds ratio, 3.5; 95% CI, 2.2-8.3; P < 0.001), pretreatment HCV RNA levels of ≤ 5 log_10 _UI/mL (odds ratio, 3.2; 95% CI, 1.2-7.2; P < 0.001), ALT levels ≥ 150 U.I./L (odds ratio, 3.1; 95% CI, 1.4-6.8; P < 0.001), and fibrosis score ≤ 3 (odds ratio, 3.2; 95% CI, 1.3-7.5; P < 0.001) were the strongest covariates independently associated with SVR (Table 4). None of the baseline characteristics, including HCV genotype, were significantly associated with relapse.

**Table 4:** Factors independently associated with sustained virological response according to stepwise logistic regression analysis.

Variable	Coefficient β	Odds ratio (95% confidence interval)	P value
Rapid virological response	1.6	3.5 (2.2-8.3)	< 0.001

Pretreatment HCV RNA levels ≤ 5 log_10_	1.5	3.2 (1.2-7.2)	< 0.001

Pretreatment ALT levels ≥150 U.I./L	1.3	3.1 (1.4-6.8)	< 0.001

Pretreatment fibrosis score ≤3	1.5	3.2 (1.3-7.5)	< 0.001

### Safety profile

Five patients of Group A2 (7%) and 7 of Group B (10%) withdrew from the study due to different adverse effects. No patient of Group A1 withdrew from the study because of adverse effects. (p < 0.05 compared with Group B). Adverse events related to treatment in the 3 study groups are reported in the Table 5.

**Table 5:** Adverse events and dose modifications according to treatment group.

	Group A1 n = 72	Group A2 n = 71	Group B n = 67
Total Discontinuation n(%)	0	5 (7.0)	7 (10)*

-anemia	0	3	3

-neutropenia	0	1	2

-depression	0	0	1

-ulcerative colitis	0	1	0

-cough	0	0	1

			

Dose modification n(%)			

-Peginterferon	1 (1)	2 (3)	2 (3)

-Ribavirin	2 (3)	9 (12)	9 (13)

			

Adverse events n(%)			

-Anemia	5 (7)	6 (8)	6 (9)

-Neutropenia	2 (3)	1 (1)	2 (3)

-Depression	2 (3)	2 (3)	1 (1)

-Cutaneous rash	0	0	1 (1)

-Alopecia	0	1 (1)	1 (1)

-Fatigue	2 (3)	4 (5)	6 (9)

* P < 0.05 compared with Group A1.

## Discussion

Our study confirmed the hypothesis that in patients with HCV genotype 2 or 3, a strategy of variable-duration treatment with PEG-IFN-α-2a and ribavirin achieves rates of SVR similar to those achieved with standard treatment, with a low rate of relapse. According to logistic regression analysis, a RVR is an independent covariate that is predictive of a SVR, along with pretreatment HCV-RNA levels, ALT values and fibrosis scores. Moreover, in our study, treatment for 12 weeks was associated with fewer adverse effects and less withdrawals from therapy, compared with treatment for 24 weeks, without significant difference in the rate of relapse.

In the ACCELERATE study [16], the rate of SVR in patients treated with PEG-IFN-α-2a and ribavirin for 16 weeks was lower than that in patients treated for 24 weeks (62% vs 70%). But among patients with a RVR, the rate of SVR was comparable to that observed in our study (79% vs 85%) [16]. The fixed dose of ribavirin (800 mg/day) administered during the ACCELERATE study may account for the reduced rate of SVR observed in patients treated for 16 weeks, since a weight-dependent dosage of ribavirin may increase the rate of SVR in patients treated with a short-term schedule. However, our results are in agreement with those reported by von Wagner et al.[17] from a randomized study comparing 16 weeks with 24 weeks of combination therapy with PEG-IFN-α-2a plus a weight-dependent ribavirin dosage in patients infected with HCV genotype 2 or 3. In this study, combination therapy for 16 or 24 weeks achieved rates of SVR among patients with a RVR at week 4 that were similar to SVR rates reported in our study [17]. Our results are also in agreement with those reported by Mangia et al. [4] and by Dalgard et al.[18], in which a 12 to 14-week regimen of PEG-IFN-α-2b and ribavirin (adjusted for weight) was associated with a significant rate of SVR, in patients with genotype 2 or 3 who had achieved an RVR.

Although in our study the rate of response at week 4 was greater in patients with genotype 2 (55% vs 45%), the rates of SVR were similar in patients with genotype 2 or 3 who had an RVR and who were treated for either 12 or 24 weeks. This finding is in agreement with that reported by Mangia et al.[4] and by Neumann et al. in the DITTO study [14] where the role of genotype appears to be relatively small after early viral clearance. However, among patients who did not achieve an RVR and who were treated for 24 weeks, the rate of SVR was higher, although not statistically significant, among those with HCV genotype 2 than among those with genotype 3 (60% vs 40%).

In our study we have observed a slight increase of SVR in patients of Group A1 compared with Group A2, however, without a difference statistically significant. A higher incidence of cirrhotic patients in group A2 and more frequent ribavirin dose modification could explain this behavior. In the study of Lagging et al. [19] treatment for 12 weeks in HCV infection with genotype 2 or 3 was inferior to 24 weeks of treatment. But 53% of all patients in this study had bridging fibrosis or cirrhosis compared with only 18% in the study of Mangia et al [4], 23% in that of Dalgard et al.,24% in the ACCELERATE trial and 29% in our study. Moreover, in the study of Lagging et al., moderate or severe hepatic steatosis was present in 26% of all the patients and this could have contributed as cofactor in non response to antiviral treatment and in a reduced rate of SVR.

## Conclusions

In conclusion, our findings suggest that patients with HCV infection with genotype 2 or 3 who have undetectable HCV-RNA levels after 4 weeks of treatment with PEG-IFN-α-2a and ribavirin may achieve high virological response rates with 12 weeks of therapy and do not require 24 weeks of treatment, especially if they have low HCV-RNA levels, high ALT levels, and a low fibrosis score at baseline. In fact, in this subset of patients, short-term therapy may improve the clinical outcome of HCV with fewer adverse effects related to long-term therapy.

## Abbreviations

HCV: hepatitis C virus; PEG-IFN-α: pegylated interferon alfa; ETR: end of treatment response; SVR: sustained virological response; RVR: rapid virological response; ALT: alanine aminotransferase; CLEO: Club Epatologi Ospedalieri; HBV: hepatitis B virus; HDV: hepatitis delta virus; HIV: human immunodeficiency virus; HAI: histological activity index; CI: confidence interval.

## Competing interests

All the Authors declares the they not have received reimbursements, fees, funding, or salary from an organization that may in any way gain or lose financially from the publication of this manuscript, either now or in the future. All the Authors declares that they do not hold any stocks or shares in an organization that may in any way gain or lose financially from the publication of this manuscript, either now or in the future. All the Authors declares that they do not hold or apply any patents relating to the content of the manuscript. All the Authors declares that they have not received reimbursements, fees, funding, or salary from an organization that holds or has applied for patents relating to the content of the manuscript. All the Authors declares that they not have any other financial competing interests.

For all the Authors

Adriano M Pellicelli MD

## Authors' contributions

FM Concept-Design-Manuscript editing-Literature search, AMP Concept-Design-Manuscript editing-Manuscript review-Manuscript preparation, MR Literature search-Data acquisition-Design, GB Manuscript review-Manuscript editing-Statistical analysis-Data acquisition-Data analysis, AB Manuscript preparation-Literature search, EM Data acquisition-manuscript preparation, MEB Data acqusition-Literature search, LN Manuscript review-Data acquisition, PA Data acquisition, GB Concept-Manuscript editing, CD Manuscript editing-Data acquisition, AP Data acquisition-Manuscript editing, AnP Data acquisition-Manuscript review, AA Manuscript review-Data acquisition, FS Manuscript editing-Definition of intellectual concept

All The Authors read and approved the final manuscript

## Pre-publication history

The pre-publication history for this paper can be accessed here:

http://www.biomedcentral.com/1471-230X/10/21/prepub
